# Efficacy of Ibuprofen Lysine on First-Trimester AbortionRelated Pain and Hemorrhage: A Randomized TripleBlinded Clinical Trial

**DOI:** 10.34172/aim.2023.32

**Published:** 2023-04-01

**Authors:** Zahra Najmi, Atousa Dabiri Oskoei, Shabnam Tofighi, Hamideh Gholami, Lida Garrosi, Faranak Amini

**Affiliations:** ^1^Department of Obstetrics and Gynecology, Zanjan University of Medical Sciences, Zanjan, Iran; ^2^Department of Obstetrics and Gynecology, Mousavi Hospital, Zanjan University of Medical Sciences, Zanjan, Iran; ^3^Department of Oncology, Mousavi Hospital, Zanjan University of Medical Sciences, Zanjan, Iran; ^4^Department of Obstetrics and Gynecology, Isfahan University of Medical Sciences, Isfahan, Iran

**Keywords:** Abortion, Hemorrhage, Ibuprofen Lysine, Pain

## Abstract

**Background:**

Some recent trials have reported high efficacy for nonsteroidal anti-inflammatory drugs (NSAIDs) in relieving medical abortion-related pain. The aim of this study was to determine the beneficial effect of oral NSAIDs (ibuprofen lysine) in reduction of pain and hemorrhage in first-trimester medical abortion.

**Methods:**

This randomized triple-blinded clinical trial was performed on 98 pregnant women who were candidate for medical abortion within the first-trimester period (gestational age<12 weeks). The participants were randomly assigned to receive ibuprofen lysine (684 mg orally every 4 hours) or placebo. All patients were initially treated with misoprostol (800 µg every 3 hours). Pain intensity and rate of hemorrhage were assessed every hour up to 15 hours after receiving the first dose of misoprostol by visual analogue scaling (VAS) and pictorial blood loss assessment chart (PBAC), respectively.

**Results:**

Assessing the mean pain score within 15 hours of receiving misoprostol showed significantly lower pain intensity within the first 10 hours of assessment in the group receiving NSAID in comparison with the control group (*P*<0.001). The bleeding rate was also significantly lower in the NSAID group at the fifth (*P*=0.013) and ninth (*P*=0.040) hour of receiving misoprostol compared to the control group. We found no difference in abortion-related complication rate between the NSAID and placebo groups (8.3% versus 8.0%, *P*=0.952).

**Conclusion:**

The use of NSAIDs (ibuprofen lysine) is a good pharmacological analgesic option for relieving medical abortionrelated pain and hemorrhage.

## Introduction

 Induction of abortion is common in pregnant women for medical reasons related to the mother or fetus; however, it may be accompanied by some major complications including pain, bleeding, infection, and even septic shock.^1^ Abortion in the first trimester is associated with moderate pain; however, this pain may be more severe, especially with increasing gestational age.^2,3^ Medical abortion is known as a painful process, as it involves the passing of the retained pregnancy conception through the cervix and the uterine smooth muscles contraction, with about 75% of women who have experienced early medical abortion before 9 weeks using opiate-based analgesia.^4^ In two different studies by Wiebe, approximately 20% of the participants reported pain scores of 9 or 10 based on VAS, which means severe pain in medical abortion.^5,6^ In addition, several studies have reported that 80%–100% of women required the use of analgesics.^7,8^ For pain relief, there are various options; however, studies have not approved a definitive regimen.^9,10^ In spite of this issue, management nonsteroidal anti-inflammatory drugs (NSAIDs) are prescribed routinely for pain and typically initiated as pain begins.^11,12^ Although supplemental narcotics have limited value, they may be also prescribed.^13^ Various painkillers are used to relieve post-abortion pain, the most important of which are opioids. Although opioids are very effective in controlling abortion-related pain, because of their side effects such as drowsiness, nausea and vomiting, ileus, constipation, respiratory suppression, central nervous system inhibition and even addiction, researchers try to identify other analgesic medications with acceptable efficacy along with higher safety.^14^

 An ideal pain relief method for abortion has not been specified. Recently, it is reported that the use of oral and intravenous NSAIDs has been successful.^15^ NSAIDs can effectively inhibit the biosynthesis of prostaglandins through blocking some special enzymes including cyclooxygenase enzymes (COX-1 or COX-2).^16^ However, there is some evidence suggesting that the pain relieving effect of NSAIDs is unrelated to its inhibitive effects on prostaglandin synthesis. In this regard, interfering with G-protein-mediated signal transduction might form the basis of analgesia with NSAIDs.^17^

 The American College of Obstetricians and Gynecologists’ practice guideline on the management of medical abortion^18^ reported that NSAIDs do not block the action of prostaglandin receptors, although they inhibit the synthesis of new prostaglandins; therefore, they will not inhibit the prostaglandin effect which is prescribed for medical abortion. At the 2005 American College of Obstetricians and Gynecologists’ annual conference, a retrospective analysis was presented on the use of ibuprofen on 416 women who referred for medical abortion of pregnancies at ≤ 56 days of gestation, and received misoprostol after methotrexate.^19^ They concluded that using ibuprofen does not obstruct the mechanism of misoprostol as a uterine contraction inducer.

 According to the current recommendations of the World Health Organization (WHO) and other public health and professional medical associations, there is an emphasis on NSAIDs, particularly ibuprofen, as first-line treatment for pain management accompanying medical abortion.^20^ The WHO also announced that evaluation of the timing of pain medication administration, and research to inform more pain management options for medical abortion, including additional medicines, is a priority.^12^ Despite the high efficacy of NSAIDs in relieving induced abortion-related pain reported from some observations, few interventional studies have been conducted to demonstrate their analgesic effects as well as their safety in patients who are candidate for induced abortion. The aim of this study was to determine the efficacy of ibuprofen lysine on first-trimester abortion-related pain and hemorrhage.

## Materials and Methods

###  Study Population 

 This randomized triple-blinded clinical trial was performed on pregnant women who were candidate for medical abortion within the first-trimester period (gestational age < 12 weeks) referring to Ayatollah Mousavi Hospital in Zanjan in 2018. All subjects aged 18 to 40 years who met the inclusion criteria were recruited. All patients had one of the indications for medical abortion including blighted ovum or missed abortion based on gynecologist recommendation. Those with a history of drug allergy to NSAIDs, history of gastrointestinal or coagulation problems and patients with underlying chronic diseases such as cardiovascular, pulmonary, renal, or rheumatic diseases were excluded from the study. Hypertensive and diabetic patients were also not included in the present trial.

**Figure 1:**
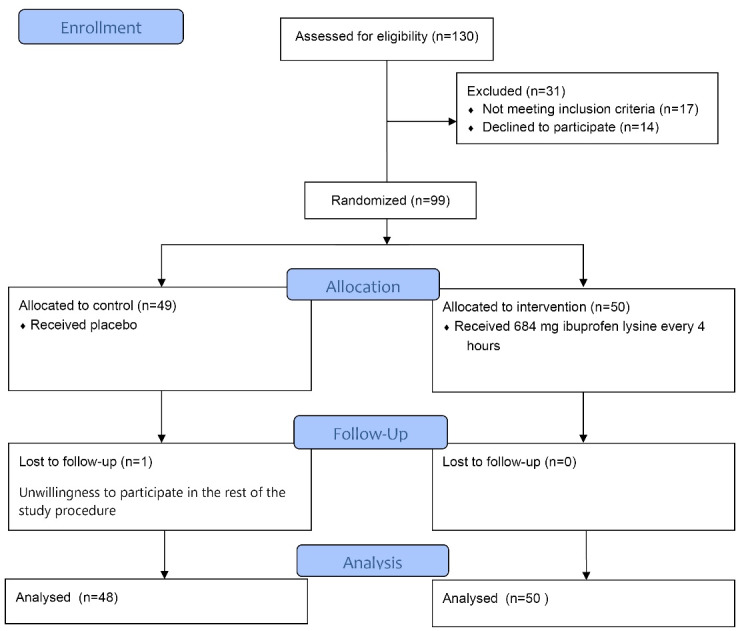
The Study Flow Chart Diagram

 Patients willing to participate in this study completed and signed the informed consent form before the study. After enrolling patients, demographic information including age, history of underlying diseases such as diabetes, hypertension, heart disease, smoking, history of gestational diseases including gestational diabetes, gestational hypertension and previous history of miscarriage were collected and recorded in the checklist (Figure 1).

###  Study Interventions 

 All patients were initially treated with misoprostol (800 µg every 3 hours). The patients were then categorized into two groups using balanced block randomization (6 blocks, 8 persons in each block) as the intervention group receiving ibuprofen lysine (684 mg orally every 4 hours) or the control group receiving placebo with the same shape and size as ibuprofen lysine tablets. Random assignmentwas performed by a physician who was unaware of the study process and did not intervene in any of the study stages. Patients were also completely unaware of the study process; however, before the intervention, the method of drug administration and the probable side effects of the drugs such as gastrointestinal bleeding, peptic ulcer, and coagulation disorders were explained for the candidate patients. Data analysis was performed by the project consultant and the project manager who were not informed about the contents; thus the trial was triple-blinded.

###  Study Assessments

 The patients were monitored by a single gynecologist during the treatment period every one hour. Before intervention, the level of pain was assessed every hour up to 15 hours after receiving the first dose of misoprostol using the visual analogue scaling (VAS) method that scores the pain from 0 (without any pain) to 10 (the most severe pain expected). The patient’s bleeding rate was assessed every hour up to 15 hours after receiving the first dose of misoprostol and recorded based on the pictorial blood loss assessment chart (PBAC). In this method, the number of tampons or towels used and the degree to which they are stained with blood are recorded. In PBAC, the patient receives a score of 5, 1, 0 and 20 based on the amount of bleeding, and at the end, according to the number of sanitary pads used, the total score is determined and the severity of bleeding is estimated. Blood pressure was measured on the right arm using a digital sphygmomanometer with a Novin S100 monitor while lying. Heart rate and respiratory rate were also determined using the Novin S100 monitoring system. Body mass index (BMI) was calculated by dividing weight by height squared. Patient was continued for up to 6 hours after delivery of pregnancy products and lasted up to 15 hours. In case of non-excretion of pregnancy products within 15 hours from the beginning of the initial intervention for abortion, the patient was excluded from the study. The primary endpoint was to assess pain intensity before and after medication in the intervention and placebo groups. The secondary endpoint was to assess and compare time to start analgesia after drug treatment, the prevalence rate of intervention-related complications such as hemodynamic instability, postoperative nausea and vomiting or loss of consciousness, the rate of requiring emergency surgery, and the bleeding rate.

###  Statistical Analysis

 For statistical analysis, results were presented as mean ± standard deviation (SD) for quantitative variables, and frequency (percentage) for categorical variables. Kolmogorov–Smirnov test was used for checking the normality of data. Continuous variables were compared using independent *t *test or Mann-Whitney test whenever the data did not appear to have normal distribution or when the assumption of equal variances was violated across the study groups. Categorical variables were, on the other hand, compared using chi-square test. For statistical analysis, the statistical software SPSS version 23.0 for Windows (IBM, Armonk, New York) was used.

## Results

 In total, 98 women candidate for medical abortion were stratified into two interventional (n = 48) and placebo (n = 50) groups. As shown in Table 1 and according to baseline characteristics, no significant difference was found between the two groups in mean age, mean BMI, mean gestational age, type of abortion (blighted ovum, missed abortion, legal abortion), history of abortion or gravidity.

**Table 1:** Baseline Characteristics in the Two Study Groups

**Characteristic **	**Intervention Group**	**Placebo Group**
Mean age, year	28.39 ± 5.64	27.60 ± 6.53
Mean body mass index, kg/m^2^	26.14 ± 4.12	25.13 ± 3.74
Mean gestational age, week		
Based on LMP	10.33 ± 1.61	10.18 ± 1.76
Based on sonography	7.18 ± 1.81	7.62 ± 1.78
Type of abortion		
Blighted ovum	31 (64.6)	38 (76.0)
Missed abortion	16 (33.3)	12 (24.0)
Legal abortion	1 (2.1)	0 (0.0)
History of abortion	14 (29.2)	6 (12.0)
History of vaginal delivery	21(43.8)	21 (42.0)

LMP, last menstrual period

**Figure 2:**
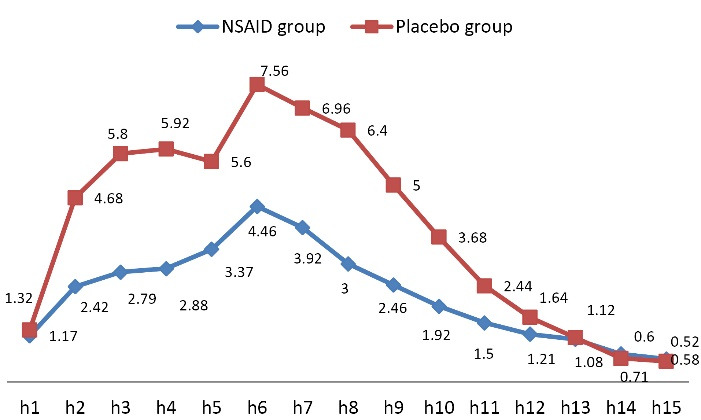
Trend of Change in Pain Intensity Score

**Figure 3:**
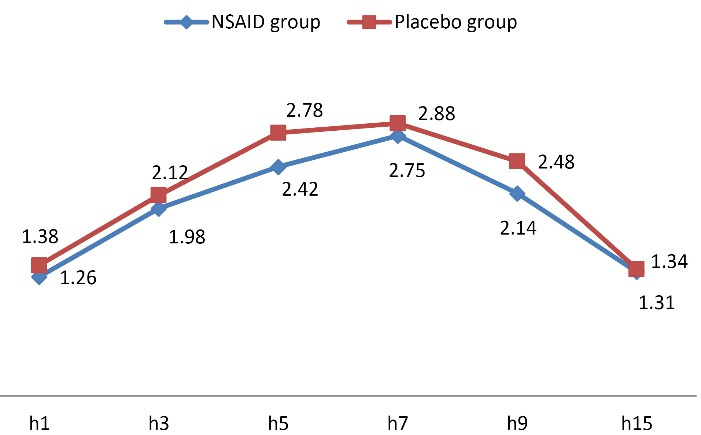
Trend of Change in Level of Hemorrhage

 Assessing the mean pain score within 15 hours of receiving misoprostol showed significantly lower pain intensity within the first 10 hours of assessment in the group receiving NSAID compared to the control group (*P* < 0.001) (Figure 2 and Table 2). The rate of bleeding was also significantly lower in the NSAID group at the fifth (*P* = 0.013) and ninth (*P* = 0.040) hour of receiving misoprostol compared to the control group (Figure 3 and Table 3).

**Table 2:** Comparison of Pain Intensity Between the Intervention and Control Groups

**Variable**	**Hour**	**Intervention Group**		**Control Group **		***P*** ** Value **
		**Number**	**Mean±SD**	**Number**	**Mean±SD**	
Pain intensity	1	48	1.17 ± 2.06	50	1.32 ± 2.16	0.720
	2	48	2.42 ± 2.18	50	4.68 ± 3.27	**<0.001**
	3	48	2.79 ± 2.36	50	5.80 ± 2.98	**<0.001**
	4	48	2.83 ± 2.25	50	5.92 ± 2.97	**<0.001**
	5	48	3.37 ± 2.55	50	5.60 ± 3.16	**<0.001**
	6	48	4.46 ± 2.19	50	7.56 ± 2.84	**<0.001**
	7	48	3.92 ± 2.54	50	6.96 ± 3.31	**<0.001**
	8	48	3.00 ± 2.70	50	6.40 ± 3.31	**<0.001**
	9	48	2.46 ± 2.55	50	5.00 ± 3.31	**<0.001**
	10	48	1.92 ± 2.29	50	3.68 ± 3.22	**0.002**
	11	48	1.50 ± 2.20	50	2.44 ± 3.16	0.090
	12	48	1.21 ± 2.47	50	1.64 ± 2.72	0.411
	13	48	1.08 ± 2.54	50	1.12 ± 2.33	0.941
	14	48	0.71 ± 1.96	50	0.6 ± 1.52	0.760
	15	48	0.58 ± 1.93	50	0.52 ± 1.26	0.848

**Table 3:** Comparison of the Level of Hemorrhage in the Intervention and Control Groups

**Variable**	**Hour**	**Intervention Group**		**Control Group **		***P*** ** Value **
		**Number**	**Mean±SD**	**Number**	**Mean±SD**	
Level of hemorrhage	1	48	1.46 ± 0.74	50	1.38 ± 0.53	0.548
	3	48	1.98 ± 0.76	50	2.12 ± 0.77	0.365
	5	48	2.42 ± 0.68	50	2.78 ± 0.74	**0.013**
	7	48	2.75 ± 0.73	50	2.88 ± 0.74	0.394
	9	48	2.14 ± 0.82	50	2.48 ± 0.76	**0.040**
	15	48	1.31 ± 0.72	50	1.34 ± 0.69	0.847

 We found no significant difference in abortion-related complication rate between the NSAID and placebo groups (8.3% versus 8.0%, *P* = 0.952).

Tables 4 to 6 summarize the impact of baseline factors (patient’s age, BMI and history of abortion) on pain severity in the two groups receiving NSAID and placebo. In this regard, we first observed that in the group receiving NSAID, obese women experienced higher pain intensity, especially in the last hours of evaluation compared to the non-obese. Second, pain intensity in both groups was independent from age or history of abortion.

**Table 4:** Pain Severity According to Patient’s Age

**Group **	**Time**	**Pain score (Mean±SD)**		***P*** ** Value**
		**<20 years** **(n=31)**	**>30 years** **(n=17)**	
NSAID group	First 4 hours	2.09 ± 1.68	2.68 ± 1.89	0.280
	Second 4 hours	3.43 ± 1.70	4.15 ± 2.22	0.221
	Third 4 hours	1.73 ± 2.01	1.85 ± 1.98	0.834
	Last 3 hours	0.71 ± 2.02	0.94 ± 1.99	0.704
Placebo group	First 4 hours	4.30 ± 2.18	4.67 ± 2.60	0.595
	Second 4 hours	6.80 ± 2.09	6.33 ± 2.24	0.467
	Third 4 hours	3.56 ± 2.55	2.53 ± 2.34	0.136
	Last 3 hours	0.73 ± 1.58	0.78 ± 1.79	0.921

**Table 5:** Comparison of Pain Severity According to Patient’s Body Mass Index Between the Intervention and Control Groups

**Group**	**Time**	**Pain score**			***P*** ** Value**
		**BMI**			
		**<20** **(n=2)**	**20 to 30** **(n=38)**	**>30** **(n=6)**	
NSAID group	First 4 hours	2.25 ± 1.06	2.42 ± 1.86	1.75 ± 1.63	0.704
	Second 4 hours	3.75 ± 2.47	3.59 ± 1.71	4.58 ± 3.12	0.518
	Third 4 hours	1.75 ± 1.06	1.38 ± 1.74	3.92 ± 2.67	0.013
	Last 3 hours	1.33 ± 1.88	0.37 ± 1.20	3.22 ± 4.12	0.003
Placebo group	First 4 hours	7.00 ± 1.73	4.19 ± 2.18	3.87 ± 2.50	0.121
	Second 4 hours	8.50 ± 0.50	6.54 ± 2.08	5.87 ± 3.27	0.521
	Third 4 hours	2.83 ± 2.02	3.21 ± 2.55	3.62 ± 3.30	0.889
	Last 3 hours	0.00 ± 0.00	0.82 ± 1.69	1.17 ± 2.33	0.657

**Table 6:** Comparison of Pain Severity According to History of Abortion Between the Intervention and Control Groups

**Group**	**Time**	**Pain Score**		***P*** ** Value**
		**History (-)** **(n=34)**	**History (+)** **(n=14)**	
NSAID group	First 4 hours	2.21 ± 1.27	2.54 ± 2.64	0.662
	Second 4 hours	3.51 ± 1.76	4.11 ± 2.25	0.335
	Third 4 hours	1.88 ± 1.95	1.50 ± 2.10	0.549
	Last 3 hours	0.78 ± 2.09	0.81 ± 1.79	0.969
Placebo group	First 4 hours	4.42 ± 2.31	4.50 ± 2.64	0.938
	Second 4 hours	6.68 ± 2.08	6.25 ± 2.70	0.647
	Third 4 hours	3.17 ± 2.47	3.33 ± 3.03	0.883
	Last 3 hours	0.73 ± 1.71	0.89 ± 1.17	0.824

## Discussion

 Due to high efficacy of NSAIDs in terms of their anti-inflammatory and analgesic effects, various types of these drugs have been employed in both pregnancy-related pain as well as pain related to medical abortion. In line with previous trials, we aimed to assess the analgesic effects of ibuprofen lysine as a common NSAID used in different clinical settings in relieving abortion-related pain. We found higher efficacy of this drug on relieving post-abortion pain (within 10 hours of administrating misoprostol) compared to placebo. Due to the fact that abortion is an inflammation-based process, the use of NSAIDs can not only inhibit the inflammatory cascade and thus accelerate post-abortion recovery, but can also effectively reduce pain intensity as well as reduce the likelihood of menorrhagia.^21^ This effectiveness has been also demonstrated in similar trials. In a systematic review by Jackson and Kapp in 2020,^15^ compared with placebo, the prophylactic use of NSAIDs could decrease pain severity as well as additional opioid requirements in women who were scheduled for medical or surgical termination of pregnancy. More interestingly, in their study, paracervical block was not significantly effective. In a clinical trial by Livshits et al,^22^ compared to paracetamol as a common analgesic drug used among experts for medical abortion, ibuprofen was more effective in relieving abortion-related pain. However, some other types of NSAIDs, even recent generations, were not helpful in abortion pain relief. As indicated by Tintara et al in 2018^23^ with respect to the efficacy of celecoxib for abortion pain relief, single-dose 400 mg celecoxib had a significant antipyretic effect during second trimester abortions but it had inadequate effect on pain relief. It seems that the dose, method of administration and even the underlying conditions of the patients admitted to the study can all be influential factors in the effectiveness of these drugs on the pain caused by medical abortion.

 We also demonstrated high effectiveness of NSAIDs on preventing menorrhagia. The effect of these drugs on preventing menorrhagia in different stages of pregnancy (not only in abortion) has been well understood. In other words, NSAIDs have been very helpful for managing menorrhagia by reducing menstrual blood loss. A review of the literature shows 30% to 40% reduced risk of menorrhagia following the use of different types of NSAIDs.^24-26^ It seems that the mechanisms for reducing abortion-related bleeding can be similar to those reported in the case of menorrhagia.

 As another important finding, we showed higher abortion-related pain intensity in obese versus non-obese patients. Regarding the link between obesity and abortion-related complications, contradictory findings have been published. Although no association was found in some studies between BMI and abortion complications,^27^ some others identified obesity as a main determinant for abortion-related complications.^25^ Overall, medical abortion does not seem to be the preferred option for women with morbid obesity.

 In conclusion, it can be suggested that the use of NSAIDs (ibuprofen lysine) is a good pharmacological analgesic option for relieving medical abortion-related pain and hemorrhage. Such medication is preferred for non-obese patients because of the higher likelihood of painful abortion in obese women.
